# Repurposing of FDA-Approved Antiviral Drugs Against Monkeypox Virus: Comparative In Vitro Screening and Structure Based In Silico Studies

**DOI:** 10.3390/ph18121857

**Published:** 2025-12-05

**Authors:** Yassmin Moatasim, Omnia Kutkat, Mokhtar Gomaa, Yaseen A. M. M. Elshaier, Mina Nabil, Ahmed A. El-Rashedy, Wael H. Roshdy, Ghazi Kayali, Mohamed Ahmed Ali, Rabeh El-Shesheny

**Affiliations:** 1 Center of Scientific Excellence for Influenza Viruses, National Research Centre, Giza 12622, Egypt; yasmin.moatasim@human-link.org (Y.M.);; 2 Department of Organic and Medicinal Chemistry, Faculty of Pharmacy, University of Sadat City, Sadat City 32897, Egypt; 3 Chemistry of Natural and Microbial Products Department, National Research Centre, Giza 12622, Egypt; 4 Central Public Health Laboratory, Ministry of Health and Population, Cairo 11843, Egypt; 5 Africa Centres for Disease Control and Prevention (Africa CDC), Haile Garment Square, Addis Ababa P.O. Box 3243, Ethiopia; 6 Human Link, Dubai P.O. Box 48800, United Arab Emirates

**Keywords:** Monkeypox, in silico, drug repositioning, molecular docking, antiviral screening

## Abstract

**Background/Objectives**: Monkeypox is endemic to the African continent and has recently garnered global attention due to reported outbreaks in non-endemic nations. No approved drug is available for non-severe cases, and some isolates gained resistance to approved antivirals. In this study, we employed a drug repositioning strategy to evaluate the efficacy of existing FDA-approved antiviral drugs if repurposed for use against emerging Monkeypox, representing a cost-effective method for identifying novel therapeutic interventions. **Methods**: Methodology including Egyptian virus strain isolation, propagation and titration followed by in vitro studies, molecular docking and molecular dynamics simulations combined with binding free energy were carried out. Twenty-three FDA-approved drugs, including Abacavir, Acyclovir, Amantadine, Chloroquine, Daclatasvir, Dolutegravir, Entecavir, Favipiravir, Hydroxychloroquine, Lamivudine, Molnupiravir, Nevirapine, Oseltamivir, Penciclovir, Remdesivir, Ribavirin, Sofosbuvir, Tenofovir, Valaciclovir, Valganciclovir, Velpatasvir, Zanamivir, and Zidovudine, were screened for potential anti-monkeypox activity in vitro. In silico studies were carried out against three monkeypox proteins, Thymidylate Kinase, A42R Profilin-Like Protein, and VACV D13, to identify their potential targets. **Results**: In vitro testing showed that two antiviral drugs are positive. The employed computational methods indicate that remdesivir demonstrated superior binding patterns with elevated scores and stable complexes throughout the simulation. **Conclusions**: Our findings showed that Remdesivir therapeutic compound is potent against the tested strain of MPXV, and exhibited a robust binding affinity for Thymidylate Kinase, A42R Profilin-Like Protein, and VACV D13 enzymes, and thus may potentially be utilized as antiviral for the treatment of monkeypox virus.

## 1. Introduction

Monkeypox (Mpox) is an emerging zoonotic disease that results in a smallpox-like illness in humans, caused by the monkeypox virus (MPXV), a species of the Orthopoxvirus genus of the Poxviridae family. MPXV is an enveloped virus with a linear double-stranded DNA genome of around 200 kb encoding ~190 non-overlapping open reading frames (ORFs) [1]. The MPXV was first isolated from laboratory research monkeys in 1958 in Denmark [2]. The first human infection was reported in 1970 in a 9-month-old boy in the Democratic Republic of the Congo [3]. Despite the nomenclature, the original source of the virus remains unknown; however, the virus is believed to be maintained in nature by animal reservoirs in rodents and non-human primates especially in Central and West Africa [4]. Human infections occur via animal bites, consuming dead animals, or direct contact with bodily fluids [5].

Phylogenetically, MPXV is classified into two distinct clades, the Congo Basin clade (clade I) originating from Central Africa and the West African clade (clade II). Clade I infections are more pathogenic than clade II, with a case fatality rate of approximately 10.6%, mainly caused by animal-to-human transmission, with limited human-to-human transmission [6]. Lower severity and mortality rates have been reported in recent clade Ib infections. In contrast, clade II is associated with milder infections and a lower case fatality rate ranging from 0.1% to 3.6% [7]. Clade II is divided into clade IIa which is zoonotic and endemic to west Africa, and clade IIb which has been recently spreading globally via human-to-human transmission and is associated with a survival rate exceeding 99% of infected cases [6].

In May 2022, an outbreak of clade IIb (B.1linage) was detected in Europe among patients with no link of traveling to endemic areas, and by June 2022, more than 1000 cases were reported in 29 non-endemic countries around the world [8]. On 26 July 2022, Mpox was declared as public health emergency of international concern (PHEIC) by the World Health Organization (WHO) [9]. On 29 August 2023, the cumulative cases of Mpox reached 89,596, including 157 fatalities, reported in 114 countries worldwide [10]. On 11 May 2023, WHO declared the end of Mpox PHEIC. A distinct and less frequent co-circulating lineage of A.2, a descendant of lineage A clade IIb, was detected in a small number of samples in Africa, India, Thailand, US, UK, and other countries. This lineage is characterized by specific genetic variations and a distinct evolutionary history compared to the more prevalent B.1 lineage responsible for the 2022 outbreak [11].

In Egypt, according to the Africa Center for Disease Control and Prevention, four laboratory-confirmed Mpox cases were reported in September and December 2022 out of 15 suspected cases, with no associated deaths [12,13]. Three viral genome sequences from these cases were published on Global Initiative on Sharing All Influenza Data (GISAID). The first isolated case belongs to lineage A.2.1, while the two other sequences belong to B.1 lineage of 2022.

According to the WHO, there is no FDA-approved antiviral treatment specific to Mpox. For individuals with high risk of exposure, administration of two doses of JYNNEOS bivalent vaccine is recommended [14]. For infected patients without severe symptoms, standard management is limited to supportive care and pain killers. However, in severe cases such as those involving immunocompromised patients, treatment with Tecovirimat is recommended [15]. Tecovirimat targets the p37 envelope protein (encoded by the *F13L* gene), involved in the viral maturation and formation of enveloped virus [16]. Tecovirimat enhanced the survival rates in animal models infected with MPXV lineage responsible for the 2022 outbreak, compared to the placebo [15,17]. In randomized clinical trials, it was proven to be safe, but it did not reduce lesion resolution time in clade I or clade II patients compared to placebo [18,19]. Moreover, cases of genotypic and phenotypic resistance to Tecovirimat have been reported underscoring the need for alternative drugs. Other possible treatments include Vaccinia Immune Globulin Intravenous (VIGIV), cidofovir, or its prodrug Brincidofovir [15,20]. Cidofovir and Brincidofovir inhibit the viral DNA polymerase encoded by *E9L* gene in various *Orthopoxviridae* viruses [21,22]. Brincidofovir is approved for the treatment of human smallpox and may be used to treat severe Mpox cases ineligible for Tecovirimat and/or severely immunodeficient. Cidofovir was approved for cytomegalovirus (CMV) treatment and was proven to be effective in vitro and in test animals against Mpox, with no supportive clinical data.

This highlights the need for developing new or repurposed antiviral agents against MPXV. Drug repurposing is an appealing approach for rapid screening of previously FDA approved antiviral drugs against various viral families, especially in emergency situations. This strategy provides shorter development times as safety, efficacy, and potential side effects have already been established.

While drug repurposing offers a rapid path to therapeutic discovery, its success hinges on a plausible biological rationale. A central question in this study involves the potential efficacy of nucleoside analogs like Remdesivir and Molnupiravir—developed against RNA viruses—against the double-stranded DNA MPXV. The hypothesis that such compounds could inhibit MPXV does not necessarily rely on the inhibition of an RNA-dependent RNA polymerase, which MPXV lacks. Instead, it is predicated on two non-mutually exclusive possibilities: firstly, the off-target inhibition of viral DNA replication machinery: Nucleoside analogs can be promiscuous. For instance, the active triphosphate form of Remdesivir (RDV-TP) is an adenosine analog. It is plausible that MPXV DNA polymerase or other nucleotide-processing enzymes (like Thymidylate Kinase) might mistakenly incorporate RDV-TP or other analog triphosphates into the growing DNA chain, leading to chain termination or mutagenesis. This phenomenon has precedent; Cidofovir, a nucleotide analog approved for DNA viruses, is also active against poxviruses by targeting their DNA polymerase [23].

Secondly, the inhibition of host or viral factors essential for replication: The antiviral effect may be indirect. MPXV replication requires host cell resources, and nucleoside analogs can disrupt host nucleotide pools or interact with host proteins involved in viral replication. Furthermore, as our in silico analysis demonstrates, these drugs may exert their effect by binding to and inhibiting crucial viral structural or regulatory proteins (like A42R or D13), a mechanism distinct from polymerase inhibition.

Therefore, this study was designed to empirically test the anti-MPXV activity of a broad panel of antivirals in vitro, followed by computational analysis to identify potential non-canonical protein targets that could explain the observed effects.

## 2. Results

### 2.1. Cytotoxicity and Antiviral Assay

**Figure 1:**
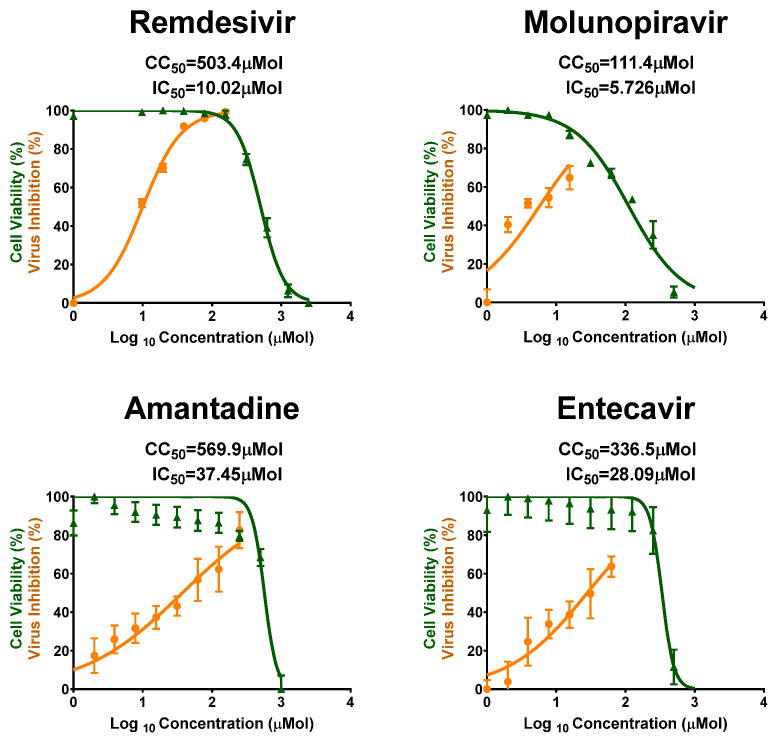
CC50 and IC50 values of the most promising antivirals against MPXV as tested in vitro in Vero-E6 cells. Values of CC50 and IC50 were calculated using non-linear regression analysis by plotting log inhibitor versus normalized response (variable slope) with Graph Pad Prism software (version 8.0.2); All values are listed in Table 1.

**Figure 2:**
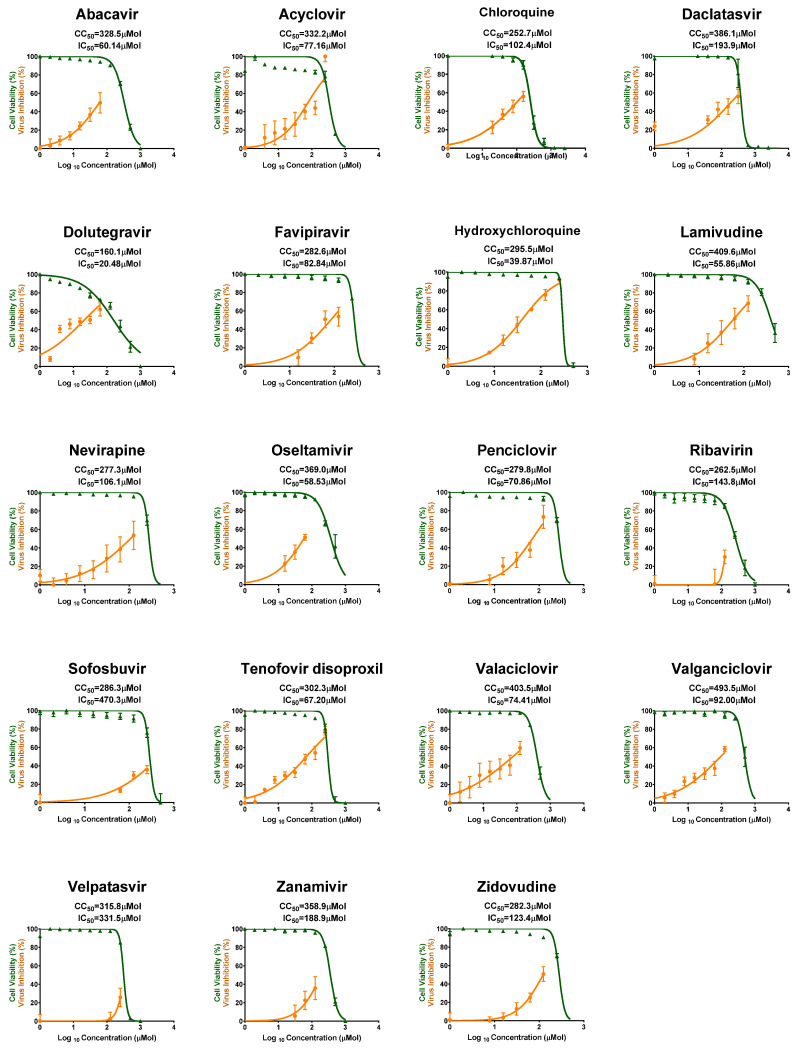
CC50 and IC50 of the other tested antiviral drugs against MPXV in Vero-E6 cells. These drugs showed moderate to no antiviral activity. All values are listed in Table 1.

Vero-E6 cells were used for the screening of antiviral activity of the 23 tested FDA-approved antiviral drugs against MPXV in vitro, using crystal violet staining mechanism to assess the cytotoxicity concentration (CC50), half maximal inhibitory concentration (IC50), and selectivity safety index (SI = CC50/IC50). Results are shown in Figure 1 and Figure 2, and in Table 1. Remdesivir showed the most promising and highest antiviral activity against MPXV, with IC50 = 10.02 µMol and SI = 50.24, followed by Molnupiravir with IC50 = 5.726 µMol and SI = 19.455, Amantadine with IC50 = 37.45 µMol and SI = 15.2, and Entecavir with IC50 = 28.09 µMol and SI = 11.98, as shown in Figure 1.

Some antivirals showed moderate antiviral activity such as Dolutegravir, Hydroxychloroquine, and Lamivudine, while the rest of the tested antivirals showing low to no activity against MPXV (Figure 2).

### 2.2. Mechanism of the Binding Interaction Based on the Computation of the Binding Free Energy

A popular technique for determining the free binding energies of small molecules to biological macromolecules is the molecular mechanics energy approach (MM/GBSA), which combines the generalized Born and surface area continuum solvation and can be more accurate than docking scores [24]. The MM-GBSA technique in AMBER18 was used to calculate the binding free energies by capturing snapshots of the systems’ trajectories. Table 2 shows that all the reported computed energy components (with the exception of ΔG_solv_) had high negative values, indicating positive interactions.

An in-depth analysis of each individual energy contribution, which results in the reported binding free energies, demonstrates that the interactions between the Molnupiravir and Remdesivir compounds and most target receptor protein residues are driven by the more positive Vander Waals energy components (Table 2).

While the absolute ΔG_bind_ values calculated by MM/GBSA (Table 2) are notably high (e.g., −60.56 kcal/mol for the Remdesivir-Thymidylate Kinase complex), such large magnitudes are not uncommon in computational studies for several reasons. The method captures the total gas-phase interaction energy (ΔE_elec_ + ΔE_vdW_), which can be substantial for large, complex ligands like Remdesivir forming multiple strong electrostatic and van der Waals contacts within a charged binding pocket [25]. Furthermore, the conformational sampling is limited to the bound state, and entropy contributions are often approximated or neglected, which can result in overestimated favorability. Therefore, while the absolute values should be interpreted with caution, the MM/GBSA method remains highly reliable for the relative ranking of ligands binding to the same target [26]. In this context, the consistently more favorable ΔG_bind_ for Remdesivir across all three targets, compared to Molnupiravir, provides strong computational evidence for its superior binding affinity, which aligns with our experimental findings.

### 2.3. Identification of the Critical Residues Responsible for Ligands Binding

To identify the key residues involved in the inhibition of the target receptor by Molnupiravir and Remdesivir compounds, the total energy involved when the compounds get in contact with these enzymes was further broken down into the involvement of particular site residues.

**Figure 3:**
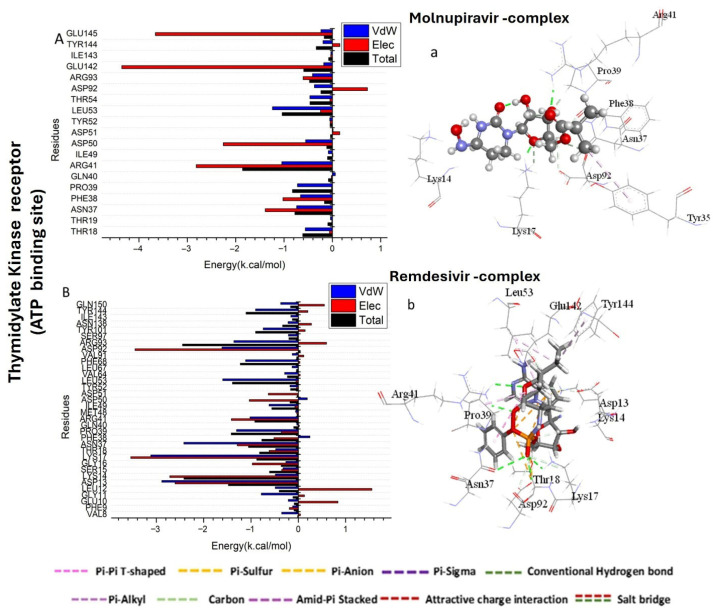

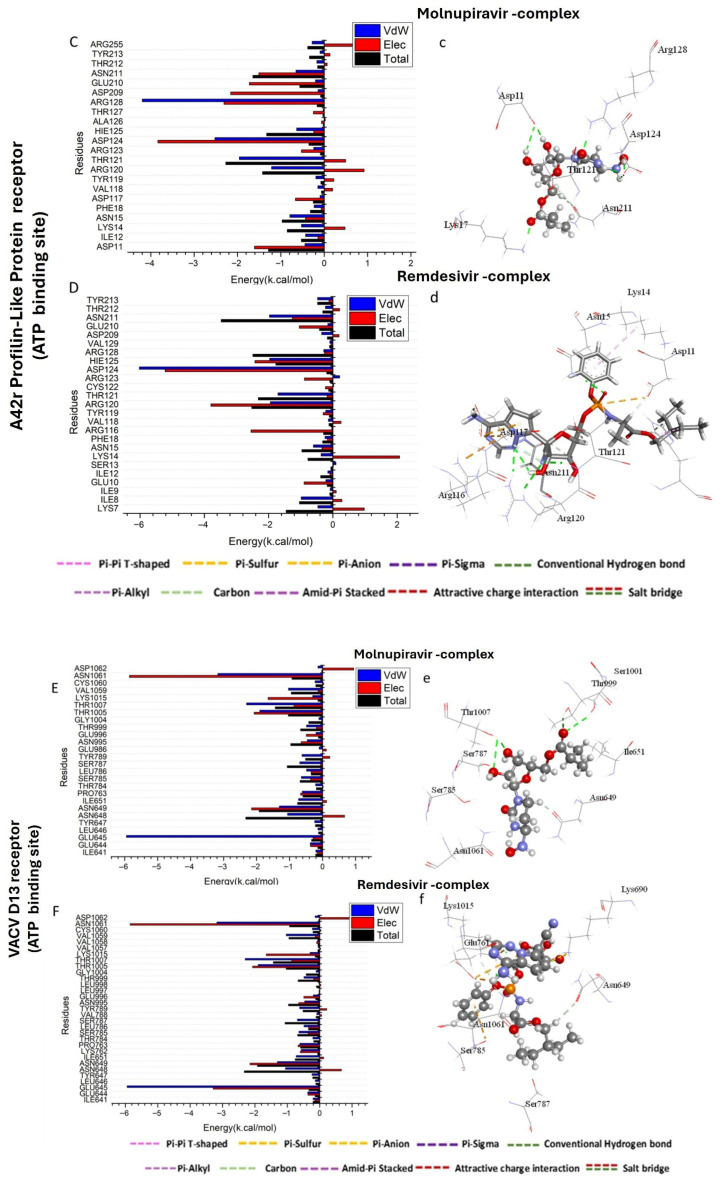
Per-residue decomposition plots showing the energy contributions to the binding and stabilization of Molnupiravir-protein binding site complex (**A**,**C**,**E**) and Remdesivir-protein binding site complex (**B**,**D**,**F**) against Thymidylate Kinase (**A**,**B**), A42R Profilin-Like Protein (**C**,**D**), and VACV D13 proteins (**E**,**F**). (**a**–**f**) shows the bonds formed in the complex formation between the tested compounds and protein active sites.

As shown in Figure 3A, the major favorable contribution of Molnupiravir compound to the Thymidylate Kinase receptor is predominantly observed from residues Arg 41 (−1.046 kcal/mol) and Leu 53 (−1.239 kcal/mol). The major favorable contribution of Remdesivir compound to the Thymidylate Kinase receptor is predominantly observed from residues Asp 13 (−2.885 kcal/mol), Lys 17 (−3.119 kcal/mol), Asn 37 (−2.419 kcal/mol), Pro 39 (−1.305 kcal/mol), Arg 41 (−1.019 kcal/mol), Leu 53 (−1.597 kcal/mol), Phe 68 (−1.113 kcal/mol), Asp 92 (−1.612 kcal/mol), Arg 93 (−1.36 kcal/mol), and Tyr 144 (−0.903 kcal/mol) (Figure 3B).

The major favorable contribution of Molnupiravir compound to the A42R Profilin-Like Protein receptor is predominantly observed from residues Arg 120 (−1.212 kcal/mol), Thr 121 (−1.955 kcal/mol), and Asp 124 (−2.518 kcal/mol) (Figure 3C). However, the major favorable contribution of Remdesivir compound to the ATP binding site receptor of A42R Profilin-Like Protein receptor is predominantly observed from residues Ile 8 (−0.985 kcal/mol), Arg 120 (−1.942 kcal/mol), Thr 121 (−1.706 kcal/mol), His 125 (−1.966 kcal/mol), and Asn 211 (−1.97 kcal/mol) (Figure 3D).

As shown in Figure 3E, the major favorable contribution of Molnupiravir compound to the VACV D13 receptor is predominantly observed from residues Glu 645 (−5.945 kcal/mol), Asn 648 (−1.059 kcal/mol), Asn 649 (−1.308 kcal/mol), Thr 1005 (−1.901 kcal/mol), Thr 1007 (−2.302 kcal/mol), Val 1059 (−1.033 kcal/mol), and Asn 1061 (−3.173 kcal/mol). However, in Figure 3F, the major favorable contribution of Remdesivir compound to the VACV D13 receptor is predominantly observed from residues Glu 645 (−4.945 kcal/mol), Asn 649 (−1.41 kcal/mol), Thr 1005 (−2.01 kcal/mol), Thr 1007 (−2.302 kcal/mol), Val 1059 (−1.203 kcal/mol), and Asn 1061 (−3.173 kcal/mol).

### 2.4. Molecular Dynamic and System Stability

**Figure 4:**
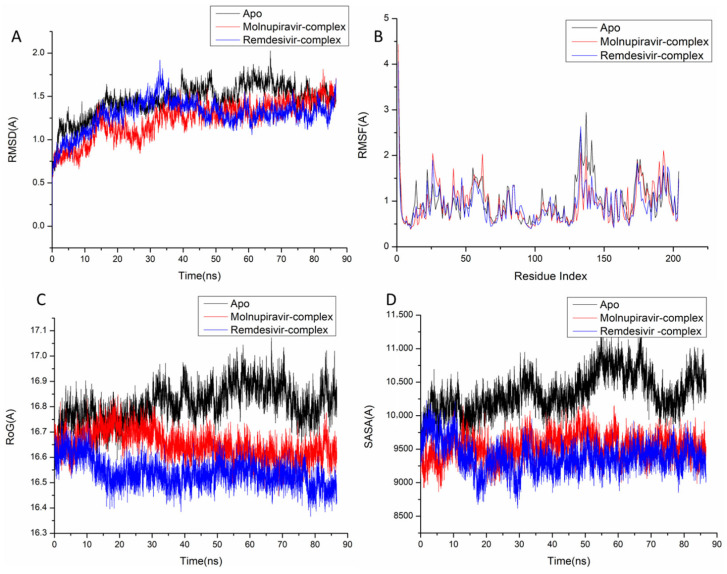
Molecular dynamic and system stability of Thymidylate Kinase complexes of Molnupiravir and Remdesivir. (**A**) RMSD of Cα atoms of the protein backbone atoms; (**B**) RMSF of each residue of the protein backbone Cα atoms of protein residues; (**C**) RoG of Cα atoms of protein residues; (**D**) solvent accessible surface area (SASA) of the Cα of the backbone atoms relative (black) to the starting minimized over 90 ns for the ATP binding site of Thymidylate Kinase receptor with Molnupiravir-complex (red) and Remdesivir-complex (blue).

**Figure 5:**
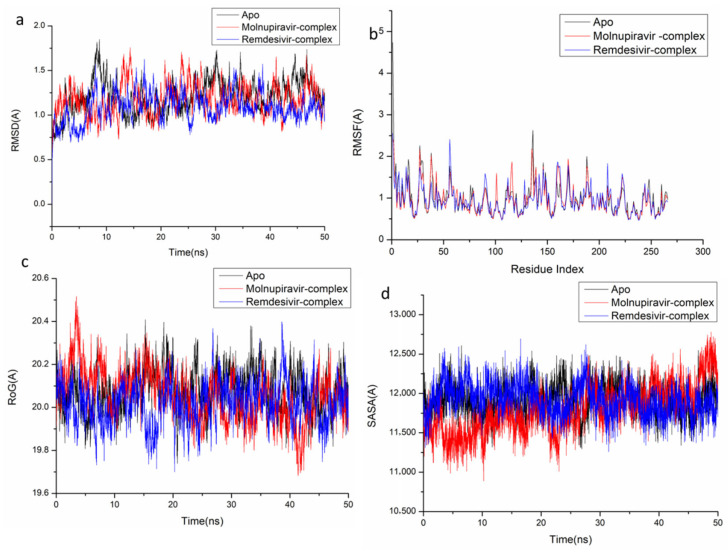
Molecular dynamic and system stability of A42R Profilin-Like Protein complexes of Molnupiravir and Remdesivir. (**a**) RMSD of Cα atoms of the protein backbone atoms; (**b**) RMSF of each residue of the protein backbone Cα atoms of protein residues; (**c**) RoG of Cα atoms of protein residues; (**d**) solvent accessible surface area (SASA) of the Cα of the backbone atoms relative (black) to the starting minimized over 50 ns for the ATP binding site of A42R Profilin-Like Protein receptor with Molnupiravir-complex (red) and Remdesivir-complex (blue).

**Figure 6:**
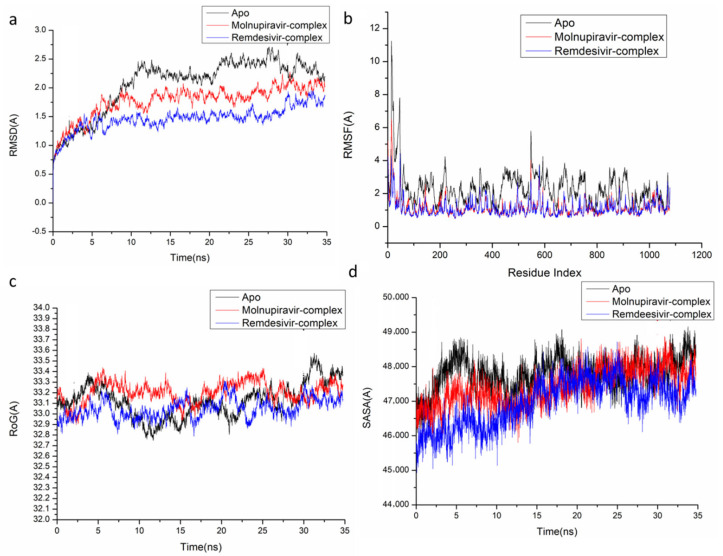
Molecular dynamic and system stability of VACV D13 complexes of Molnupiravir and Remdesivir. (**a**) RMSD of Cα atoms of the protein backbone atoms; (**b**) RMSF of each residue of the protein backbone Cα atoms of protein residues; (**c**) RoG of Cα atoms of protein residues; (**d**) solvent accessible surface area (SASA) of the Cα of the backbone atoms relative (black) to the starting minimized over 35 ns for the ATP binding site of VACV D13 receptor with Molnupiravir-complex (red), and Remdesivir-complex (blue).

The behavior of the synthesized compound upon binding to the protein’s active site as well as their interaction and stability were predicted using Molecular Dynamics (MD) modeling [27]. To identify interrupted motions and prevent any artifacts during the simulation, system stability was validated. The stability of the systems was evaluated during the simulations using Root-Mean-Square Deviation (RMSD). The recorded average RMSD values for the entire frames of the systems were 1.45 ± 0.19 Å, 1.29 ± 0.21 Å, and 1.24 ± 0.21 Å, for Apo complex, Thymidylate Kinase-Molnupiravir complex, and Thymidylate Kinase-Remdesivir complex (Figure 4A), 1.21 ± 0.18 Å, 1.17 ± 0.17 Å and 1.40 ± 0.16 Å, for Apo complex, A42R Profilin-Like Protein-Molnupiravir complex, and A42R Profilin-Like Protein-Remdesivir complex (Figure 5a), and 2.16 ± 0.2 Å, 1.77 ± 0.27 Å, and 1.46 ± 0.20 Å, for Apo complex, VACV D13-Molnupiravir complex, and VACV D13-Remdesivir complex (Figure 6a). These results indicate that, in comparison to the other systems being studied, the Remdesivir-bound to protein complex system evolved in a relatively more stable conformation.

It is necessary to assess protein structural flexibility upon ligand binding in order to examine residue behavior and its link to the ligand during MD simulation [28]. Protein residue fluctuation was evaluated using the Root-Mean-Square Fluctuation (RMSF) technique to check the effect of inhibitor binding to the respective targets throughout the simulations. The computed average RMSF values were 1.02 ± 0.49 Å, 0.96 ± 0.46 Å, and 0.91 ± 0.41 Å, for Apo complex, Thymidylate Kinase-Molnupiravir complex, and Thymidylate Kinase-Remdesivir complex (Figure 4B), 0.98 ± 0.42 Å, 0.96 ± 0.36 Å, and 0.955 ± 0.33 Å, for Apo complex, A42R Profilin-Like Protein-Molnupiravir complex, and A42R Profilin-Like Protein-Remdesivir complex (Figure 5b), and 2.31 ± 0.04 Å, 1.13± 0.58 Å, and 1.09 ± 0.47 Å, for Apo complex, VACV D13-Molnupiravir complex, and VACV D13-Remdesivir complex (Figure 6b). These results showed that, in comparison to the other systems, VACV D13-bound to protein complex system has a lower residue fluctuation.

Radius of Gyration (RoG) was discovered to evaluate the system’s overall compactness and stability upon ligand binding in MD simulation [29]. The average RoG values were 16.81 ± 0.07 Å, 16.64 ± 0.05 Å, and 16.53 ± 0.05 Å, for Apo complex, Thymidylate Kinase Molnupiravir complex, and Thymidylate Kinase-Remdesivir complex (Figure 4C), 20.07 ± 0.09 Å, 19.96 ± 0.11 Å, and 19.00 ± 0.096 Å, for Apo complex, A42R Profilin-Like Protein-Molnupiravir complex, and A42R Profilin-Like Protein-Remdesivir complex (Figure 5c), and 33.16 ± 0.82 Å, 33.20 ± 0.09 Å, and 33.04 ± 0.09 Å, for Apo complex, VACV D13-Molnupiravir complex, and VACV D13-Remdesivir complex (Figure 6c). According to the observed behavior, remdesivir-compound has a highly rigid structure against the target receptor.

The compactness of the protein hydrophobic core was examined by calculating the protein’s solvent accessible surface area (SASA). This was performed by measuring the surface area of the protein visible to the solvent, which is important for biomolecule stability [30]. The average SASA values were 10,339.59 Å, 9530.86 Å, and 9384.48 Å, for Apo complex, Thymidylate Kinase-Molnupiravir complex, and Thymidylate Kinase-Remdesivir complex (Figure 4D), 11,919.54 Å, 11,909.50 Å, and 11,822.13 Å, for Apo complex, A42R Profilin-Like Protein-Molnupiravir complex, and A42R Profilin-Like Protein-Remdesivir complex (Figure 5d), and 47,797.73 Å, 47,429.32 Å, and 46,956.32 Å, for Apo complex, VACV D13-Molnupiravir complex, and VACV D13-Remdesivir complex (Figure 6d). The SASA finding, when paired with the observations from the RMSD, RMSF, and RoG computations, confirmed that the Remdesivir-complex system remains intact inside the catalytic domain binding site of target receptors.

### 2.5. Hydrogen Bonding

**Figure 7:**
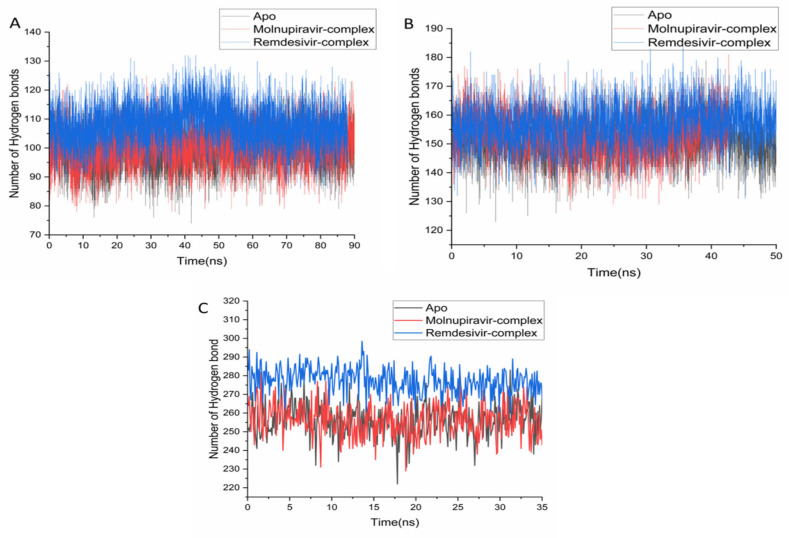
Hydrogen bond formation over time during simulation for the apo, Molnupiravir, and Remdesivir systems for 2V54 (**A**), 4QWO (**B**), and 6BED (**C**).

Hydrogen bonding is a key determinant of protein stability and ligand interactions [31]. Analysis of simulation trajectories revealed that the Remdesivir-bound system formed the highest number of persistent hydrogen bonds across all tested proteins. For the 2V54 system, Remdesivir had an average of 107.98 H-bonds, exceeding both the apo (99.89) and Molnupiravir (100.28) systems (Figure 7A). This superior H-bonding was also evident in the 4QWO system, with averages of 156.86 (Remdesivir), 151.23 (apo), and 153.21 (Molnupiravir) (Figure 7B). The most significant difference was observed for the 6BED system, where the Remdesivir complex averaged 276.96 H-bonds, more than double the apo (252.51) and Molnupiravir (254.31) systems (Figure 7C).

### 2.6. Evolution of Hydrophobic Interactions

Hydrophobic contacts provide a major driving force for ligand binding. We monitored the contact surface area between the ligands’ hydrophobic moieties and key non-polar residues over the simulation time.

The analysis revealed that Remdesivir, with its large, flexible aliphatic and aromatic segments, formed extensive and stable hydrophobic contacts. For instance, in the Thymidylate Kinase complex, Remdesivir maintained persistent contact with Leu 53 and Phe 68 (identified in Figure 3B), with the interaction surface area fluctuating minimally after the initial equilibration phase. In the VACV D13 complex, the hydrophobic packing against Val 1059 was exceptionally stable, explaining the high van der Waals (ΔE_vdW_) contribution observed in the MM/GBSA calculations (Table 2).

Molnupiravir, being a smaller and more polar molecule, was engaged in fewer and less stable hydrophobic interactions. Its contacts with analogous residues were intermittent, leading to a higher degree of fluctuation within the binding site, as reflected in its higher RMSD and RMSF values.

## 3. Discussion

Emerging and reemerging zoonotic diseases represent a major concern globally, especially in the absence of specific therapeutic agents. In recent years, MPXV clade IIb has caused a global outbreak and was declared a PHEIC by the WHO. This clade, although associated with lower mortality rates than previous clades, has spread faster and more globally in non-endemic countries with sustained human-to-human transmission.

To date, no FDA-approved antiviral treatments are available and post-exposure vaccination remains the most effective preventive measure for high-risk populations, as it was observed that lower immunity rates to smallpox would increase population susceptibility to Mpox [32]. Currently, three drugs, Tecovirimat, Cidofovir, and Brincidofovir, are authorized for Mpox treatment, though their clinical efficiency is still under investigation.

Tecovirimat showed ability to inhibit the virion envelope formation by targeting the VP37 protein in various poxviruses [18,33]). While proven safe, it is only approved for individuals at higher risk of severe disease or those with complications, and was proven to be less effective in moderate infections in clinical trials [19]. It is not approved for children or for post-exposure prophylaxis of any orthopoxvirus infection [34]. Moreover, there are reports of Tecovirimat resistance emerging in some MPXV strains. This resistance is primarily linked to mutations in the viral phospholipase *F13L* gene [35]. Cidofovir and its derivative Brincidofovir were shown to be effective against poxviruses but with not enough clinical data.

Drug repurposing is a method of identifying new therapeutic uses for previously approved drugs, that might be able to target viral machineries or cellular pathways and thus impair the ability for virus replication inside the cell. There is an urgent need for screening anti-Mpox activity of several FDA-approved antiviral drugs, which can be more effective and easily applicable in case of viral evolution or presence of resistant mutations [20].

In this study, we evaluated the anti-Mpox activity of commercially available antiviral agents through in vitro screening and in silico analysis against the clade II lineage A.2.1 MPXV isolated in Egypt. A total of 23 raw antiviral compounds were tested for anti-Mpox activity on the Vero-E6 cell line. We found that Remdesivir, a nucleoside analog that inhibits the RNA-dependent RNA polymerase (RdRp) in SARS-CoV-2, showed high efficiency in vitro to inhibit viral replication at IC50 10.02 µMol in crystal violet staining testing assay. Molnupiravir, Amantadine, and Entecavir showed moderate anti-Mpox activity in vitro. Our results support the previously published research reporting anti-Mpox activity of these compounds against MPXV clades Ib and IIb using live viral foci-inhibition assay [36]. Molnupiravir anti-Mpox activity was also supported in vitro with IC50 around 5.2 μM against Zr-599 and Liberia strains of MPXV [37].

Remdesivir and Molnupiravir were further tested in silico to determine the possible mechanism of action and drug-viral protein active site complex stability against three poxvirus viral proteins, poxvirus thymidylate kinase, A42R Profilin-like Protein from MPXV Zaire-96-I-16, and Vaccinia virus D13 protein. The MM/GBSA used for determining the free binding energies of small molecules to biological macromolecules showed that all the reported computed energy components (except for ΔG_solv_) had high negative values, indicating positive interactions, with relatively higher negative values of Remdesivir-complexes with the three proteins compared to Molnupiravir-complexes.

Through MD modeling techniques, evaluated systems stability during the simulations using RMSD showed that, in comparison to the other systems being studied, the Remdesivir-bound to protein complex systems evolved to a relatively more stable conformation. Protein residue fluctuation results indicated lower residue fluctuation of the formed complexes. RoG evaluation of the system’s overall compactness as well as its stability upon ligand binding in MD simulation showed that remdesivir has a highly rigid structure against the target receptor, and SASA finding, when paired with the observations from the RMSD, RMSF, and RoG computations, confirmed that the remdesivir-complex system remains intact inside the catalytic domain binding site of target receptors.

The most significant finding of our study is the potent in vitro anti-MPXV activity of Remdesivir, a drug formally classified as an RNA-chain terminator. This presents an apparent mechanistic paradox, as MPXV is a DNA virus. Our integrated computational and experimental approach allows us to propose several hypotheses to resolve this.

First, the most direct explanation would be the off-target inhibition of the MPXV DNA polymerase. While Remdesivir was designed for the SARS-CoV-2 RdRp, its active triphosphate form (RDV-TP) is a nucleoside analog that could potentially be recognized by the MPXV DNA polymerase. If incorporated into the viral DNA, the 1′-cyano group of Remdesivir could cause steric hindrance and lead to delayed chain termination, similar to its proposed mechanism in RNA viruses. This would align with the known activity of other nucleotide analogs like Cidofovir and Brincidofovir against poxviruses. Our molecular docking and dynamics studies against Thymidylate Kinase, a key enzyme in the dTTP synthesis pathway, provide supporting evidence for this hypothesis. The stable binding of Remdesivir to this enzyme’s active site suggests it could also disrupt the nucleotide substrate supply for the DNA polymerase, creating a dual inhibitory effect.

Second, our in silico results strongly suggest that Remdesivir’s mechanism may extend beyond nucleotide metabolism. The high-affinity, stable binding we observed to the A42R Profilin-Like Protein and the VACV D13 scaffold protein indicates that the antiviral activity could be mediated through the inhibition of viral assembly and spread. This is a structurally driven, mechanism-agnostic effect. The large, complex structure of Remdesivir may allow it to act as a molecular “wedge”, disrupting critical protein-protein or protein-lipid interactions necessary for virion morphogenesis (D13) or actin-based motility (A42R). This would represent a novel, non-polymerase mechanism of action for Remdesivir.

Third, we cannot rule out indirect effects on the host cell. Remdesivir could perturb host nucleotide pools or interact with host factors that are co-opted by the virus, thereby creating an environment less conducive to viral replication. While our study was not designed to elucidate this, it remains a plausible contributor to the overall antiviral effect.

In conclusion, the anti-MPXV activity of Remdesivir likely cannot be explained by a single mechanism. We propose that it is the result of a multi-target strategy, where its promiscuous binding capability, evidenced by our computational data, allows it to inhibit several critical points in the MPXV life cycle simultaneously—from nucleotide synthesis and DNA replication to viral assembly and cell-to-cell spread. This polypharmacology could make it particularly effective and reduce the likelihood of rapid resistance emergence. Future work should include enzymatic assays with purified MPXV DNA polymerase and Thymidylate Kinase to directly validate these hypotheses.

The analysis of chemical structures among the screened drugs provides insights into the features conducive to anti-MPXV activity. The most potent compounds, Remdesivir and Molnupiravir, share a common characteristic as nucleoside analogs, but their specific structures dictate their efficacy and potential mechanism. Remdesivir, an adenosine analog, possesses a 1′-cyano group and a phosphoramidate prodrug moiety. The 1′-cyano group is a key structural feature that may induce steric hindrance, potentially leading to delayed chain termination during viral DNA/RNA synthesis. Our in silico data supports its high-affinity binding, likely facilitated by its large, flexible structure capable of forming extensive van der Waals contacts and hydrogen bonds with multiple residues across all three target proteins (e.g., Asp 13, Lys 17 in Thymidylate Kinase; Glu 645, Asn 1061 in VACV D13). This promiscuous binding potential, driven by its complex structure, may contribute to its superior activity and high selectivity index (SI = 50.24). Molnupiravir, a ribonucleoside analog of cytidine, has a hydroxyl group as primary feature that tautomerizes between cytidine-like and uridine-like forms. This tautomerization is believed to cause lethal mutagenesis in RNA viruses. While it showed good activity (IC50 = 5.7 µM), its lower SI compared to Remdesivir and less favorable binding energies in our simulations suggest its mechanism against a DNA virus like MPXV might be different or less efficient. Its simpler structure compared to Remdesivir may limit its ability to form the stable, multi-residue interactions seen with the latter.

Other nucleoside analogs like Acyclovir and Zidovudine showed limited activity. Acyclovir’s lack of a 3′-hydroxyl group in its sugar moiety typically leads to obligate chain termination. Its poor activity here may be due to inefficient phosphorylation by MPXV kinases or poor recognition by the viral polymerase. Similarly, the azido group in Zidovudine might not be efficiently processed by the MPXV replication machinery. This highlights that not all nucleoside analogs are effective, and their activity is highly dependent on specific viral enzyme recognition and activation.

Amantadine, which showed moderate activity, has a completely different structure—a symmetric, rigid adamantane cage. Its known mechanism involves disrupting the acidification of endosomes (in influenza) or ion channel function. Its activity against MPXV suggests a potential, yet unconfirmed, host-targeted mechanism, such as disrupting viral entry or trafficking.

In conclusion, the structure-activity relationship indicates that flexible nucleoside analogs with complex modifications (like Remdesivir) are particularly promising. They appear capable of acting as substrates for viral polymerases while also potentially inhibiting other essential viral enzymes through high-affinity binding, as demonstrated in our computational models. This multi-target potential, inherent to their chemical structure, may be a critical determinant of their superior anti-MPXV efficacy.

The emergence of Mpox disease, its global outbreaks, and the lack of proven treatments prompted this study to screen FDA-approved drugs as inhibitors for MPXV. This inhibitory method seeks to curtail viral replication and growth. Molnupiravir and remdesivir exhibited favorable binding energy and enhanced affinity for the binding sites of Thymidylate Kinase, A42R Profilin-Like Protein, and VACV D13. Molecular dynamic studies further validated the structural flexibility and stability of medicines as inhibitors of Thymidylate Kinase, A42R Profilin-Like Protein, and VACV D13. Since these medications are currently sanctioned for the treatment of many human ailments, clinical studies for a new indication may proceed in accordance with FDA guidelines and authorization. This strategy may expedite the advancement of treatments for MPXV infection. The experimental tests confirm the efficacy of the recommended medications for the prevention and treatment of MPXV. Our overall data indicates that Remdesivir is potentially active in inhibiting MPXV viral replication and could be effective against other poxviruses such as vaccinia virus according to our docking study. This implies the importance of continuous screening for alternative potential antiviral agents specially for viruses with high mutation rates and possible emerging and reemerging viral variants that can skip immunity and vaccination or acquire resistance mutations. Further work is required to determine the antiviral potency against different clades of MPXV and other poxviruses. In vivo studies are also required for data validation.

## 4. Materials and Methods

### 4.1. Cells and Tested Antiviral Drugs

Vero-E6 cells were maintained, sub-cultured, and propagated in Dulbecco’s Modified Eagle Medium (DMEM) media supplemented with 10% fetal bovine serum and 1% antibiotic-antimycotic solution. Commercially available FDA-approved antiviral drugs in Egypt were collected as raw active constituent from the Egyptian Drug Authority (Cairo, Egypt) [38]. Tested antivirals (*n* = 23) in this study are listed in Table S1 with their corresponding mechanisms. Stock solutions were prepared by dissolving in dimethyl sulfoxide (DMSO, Sigma-Aldrich, Darmstadt, Germany) at a 100 µMol concentration. Table S2 lists all antivirals in this study with their metabolisms. Antiviral drugs classification based on their chemical structure is shown in Figures S2–S7. The chemical structures of all 23 tested FDA-approved drugs are provided in Supplementary Table S1 and Figure S1.

### 4.2. Virus Isolation and Propagation and Titration

MPXV strain MOH-NRC-0002/2022 (accession number OP597769) [12], clade II lineage A.2.1, was obtained from a pustule of the first Mpox case in Egypt. Virus inoculum was used to infect Vero-E6 cells in a 6-well plate at dilution 1:20 and inspected microscopically daily for the cytopathic effect (CPE) development. Harvest was tested using real-time PCR assay. Isolated virus was then subjected to plaque purification and propagation for two passages. All isolation and propagation steps were performed in Vero-E6 cell lines using DMEM media supplemented with 2% fetal bovine serum and 1% antibiotic-antimycotic solution. Propagated virus was subjected to titration by Tissue Culture Infection Dose (TCID50) assay to quantify the infectious particles.

### 4.3. Safety and Antiviral Activity by Crystal Violet Assay

The possible antiviral activity of the tested drugs against MPXV was assessed as previously described [38]. First, the CC50 of each compound was assessed in monolayers of Vero-E6 cells in 96-well plates, treated with 1:2 serial dilutions of each compound and incubated for 72 h. Fixation using 10% formaldehyde and crystal violet staining of the monolayers was performed to determine the concentration that kills 50% of treated cells (CC50), as compared to cell control. The CC50 value was calculated using GraphPad Prism software (version 8.02) by plotting log concentrations of the drugs versus the normalized response (variable slope-non-linear regression analysis).

Then the IC50 required to reduce the virus-induced CPE by 50% relative to the virus control, was assessed as follows: Set of serial dilutions of the tested drugs were prepared, mixed with 100 folds of viral TCID50, and incubated for 1 h prior to Vero-E6 cell monolayers treatment with the mix. Untreated cells that were infected with 100 TCID50 of the virus represented “virus control”, whereas cells that were not treated and not infected were the “cell control”. After 72 h of incubation at 37 °C, cells were fixed with 10% formaldehyde and stained with crystal violet. The IC50 value was calculated using non-linear regression analysis in GraphPad Prism software (version 8.02).

### 4.4. Docking Study

#### 4.4.1. System Preparation

The 3D structures of poxvirus thymidylate kinase (PDB ID: 2V54), A42R Profilin-like Protein from MPXV Zaire-96-I-16 (PDB ID: 4QWO) [39,40], and Vaccinia virus D13 protein (PDB ID: 6BED) [41] were provided by Protein Data Bank and prepared using UCSF Chimera [42]. The pH was adjusted and tuned at 7.5 using PROPKA. ChemBioDraw Ultra 12.1 was utilized to draw the extracted 2D structure. The 2D structure was optimized for energy minimization using the MMFF94 force field and the steepest descent approach in Avogadro software v1.1.0 [43], UCSF chimaera were used to eliminate hydrogen atoms prior to docking [42].

#### 4.4.2. Molecular Docking

Docking calculations were performed using AutoDock v1.5.7 [44] and Gasteiger partial charges [44,45] were allocated during docking. The AutoDock graphical user interface offered by MGL tools v1.5.7 was used to outline the AutoDock atom types. The x, y, z AutoDock Vina grid center coordinates used were 23.17, 22.70, and 5.80 for 2V54; −0.286, 5.2811, and 8.23 for 4QWO; and 85.366, 96.799, and 17.277 for 6BED. The size of the search space was set to 20 Å × 20 Å × 20 Å and exhaustiveness = 8. Docked conformations were generated in descending order according to their docking energy using the Lamarckian genetic method [44,46].

### 4.5. Molecular Dynamic

#### 4.5.1. Selection of Viral Targets for In Silico Analysis

To investigate the potential mechanism of action of the most promising antivirals identified in our in vitro screening, we selected three key MPXV proteins with essential and distinct roles in the viral life cycle: Thymidylate Kinase (A48R), A42R Profilin-Like Protein, and the Vaccinia virus D13 scaffold protein. Thymidylate Kinase (PDB: 2V54) is central to the de novo synthesis of dTTP for viral DNA replication. The A42R Profilin-Like Protein (PDB: 4QWO) is a virulence factor that facilitates actin-based motility and cell-to-cell spread, enabling the virus to evade host immunity [40]. Finally, VACV D13 (PDB: 6BED) is a critical structural component for viral morphogenesis and a validated drug target, as its inhibition by Rifampin leads to non-infectious particles [41]. This multi-target strategy allows for the evaluation of antiviral interference at the levels of genome replication, intracellular spread, and viral assembly.

#### 4.5.2. Molecular Dynamic Simulations

Exploring the physical motion of atoms and molecules is difficult to access through other methods is made possible by the incorporation of MD simulations into the study of biological systems. The knowledge gained from running this simulation offers a glimpse into the dynamic evolution of biological systems, including molecular interaction and conformational changes. The AMBER 18 package’s GPU version of the PMEMD engine was used to run the MD simulations for every system [47].

ANTECHAMBER’s General Amber Force Field (GAFF) method was used to determine each compound’s partial atomic charge [48]. Each system within an orthorhombic box of TIP3P water molecules within 10 Å of any box border was implicitly solved by the AMBER 18 package’s Leap module. Each system was neutralized by adding Na^+^ and Cl^−^ counter ions using the Leap module. Each system was first minimized in 2000 steps with a 500 kcal/mol applied restraint potential and fully minimized in 1000 steps with the conjugate gradient algorithm without restraints.

To guarantee that every system had the same number of atoms and volume, the MD simulation involved progressively heating each system from 0 K to 300 K over 500 ps. The solutes in the system were subjected to a collision frequency of 1 ps and a potential harmonic constraint of 10 kcal/mol. Each system was then heated to a constant temperature of 300 K and allowed to equilibrate for 500 ps. The number of atoms and pressure in each system were kept constant for each production simulation to model an isobaric-isothermal ensemble (NPT). The Berendsen barostat was used to keep the system’s pressure at 1 bar [49]. In each simulation, the hydrogen bond atoms were constrained using the SHAKE approach. A 2 fs step size and an SPFP precision model were incorporated into each simulation. The simulations were conducted using an NPT with randomized seeding, a Langevin thermostat with a collision frequency of 1 ps, a temperature of 300 K, a constant pressure of 1 bar, and a pressure-coupling constant of 2 ps.

#### 4.5.3. Post-MD Analysis

The CPPTRAJ [50] module of the AMBER18 suite was used to examine the trajectories after they were saved by MD simulations at 1 ps intervals. All graphs and visualizations were made using Chimera [42] and the Origin [51] data analysis tool.

#### 4.5.4. Thermodynamic Calculation

The Poisson-Boltzmann or generalized Born and surface area continuum solvation (MM/PBSA and MM/GBSA) approach has been found to be useful in estimating ligand-binding affinities [52]. The Protein-Ligand complex molecular simulations used by MM/GBSA and MM/PBSA compute rigorous statistical-mechanical binding free energy within a defined force field. The estimation of the change in binding free energy (ΔG) for each molecular species (complex, ligand, and receptor) can be represented as follows [53]:

∆G_bind_ = G_complex_ − G_receptor_ − G_ligand_ (1)

∆G_bind_ = E_gas_ + G_sol_ − TS (2)

E_gas_ = E_int_ + E_vdw_ + E_ele_ (3)

G_sol_ = G_GB_ + G_SA_ (4)

G_SA_ = γSASA (5)

The gas-phase, internal, Coulomb, and van der Waals energies are denoted by the letters E_gas_, E_int_, E_ele_, and E_vdw_. The FF14SB force field terms were used to directly evaluate the Egas. The energy involved from the polar states (GGB) and non-polar states (G) was used to calculate the solution-free energy (G_sol_). Using a water probe radius of 1.4 Å, the non-polar solvation free energy (GSA) was calculated from the Solvent Accessible Surface Area (SASA) [54,55]. On the other hand, the polar solvation (GGB) contribution was evaluated by solving the GB equation. Items S and T represent the solute’s total entropy and temperature, respectively, each residue’s contribution to the overall binding free energy was determined using Amber18′s MM/GBSA-binding free energy approach.

## Figures and Tables

**Table 1:** Results of antiviral activity of the 23 tested antiviral agents, showing the values of CC50 in µM, IC50 in µM, and SI.

Drug	M.Wt	CC50	IC50	SI
Abacavir	286.3	328.5	60.14	5.46
Acyclovir	225.2	332.2	77.16	4.31
Amantadine	187.7	569.9	37.45	15.22
Chloroquine	319.9	252.7	102.4	2.47
Daclatasvir	738.9	386.1	193.9	1.99
Dolutegravir	419.4	160.1	20.48	7.82
Entecavir	277.3	336.5	28.09	11.98
Favipiravir	157.1	282.6	82.84	3.41
Hydroxychloroquine	335.9	295.5	39.87	7.41
Lamivudine	229.3	409.6	55.86	7.33
Molnupiravir	329.3	111.4	5.726	19.46
Nevirapine	266.3	277.3	106.1	2.61
Oseltamivir	312	269	58.53	4.60
Penciclovir	253.3	279.8	70.84	3.95
Remdesivir	602.6	503.4	10.02	50.24
Ribavirin	244.2	262.5	143.8	1.83
Sofosbuvir	529.5	286.3	470.3	0.61
Tenofovir disoproxil	519.5	302.3	67.2	4.50
Valaciclovir	324.3	403.5	74.41	5.42
Valganciclovir	390.82	493.5	92	5.36
Velpatasvir	883	315.8	331.5	0.95
Zanamivir	332.2	358.9	188.9	1.90
Zidovudine	267.2	282.3	123.4	2.29

M.Wt = molecular weight.

**Table 2:** The calculated energy binding for the molnupiravir and remdesivir compounds against the catalytic binding site of target receptors.

	Energy Components (kcal/mol)				
	ΔE_vdW_	ΔE_elec_	ΔG_gas_	ΔG_solv_	ΔG_bind_
Thymidylate Kinase					
Molnupiravir-complex	−20.77 ± 0.29	−36.83 ± 0.61	−57.60 ± 0.24	41.00 ± 0.74	−16.60 ± 0.55
Remdesivir-complex	−48.35 ± 0.31	−81.92 ± 0.99	−130.27 ± 1.05	69.71 ± 0.74	−60.56 ± 0.51
A42R Profilin-Like Protein					
Molnupiravir-complex	−29.18 ± 0.31	−41.37 ± 0.89	−70.55 ± 0.90	38.78 ± 0.64	−31.77 ± 0.42
Remdesivir-complex	−51.56 ± 0.47	−56.29 ± 1.14	−107.85 ± 0.30	68.73 ± 0.89	−39.12 ± 0.65
VACV D13					
Molnupiravir-complex	−36.81 ± 0.27	−7.95 ± 0.67	−44.76 ± 0.75	11.41 ± 0.53	−33.34 ± 0.42
Remdesivir-complex	−40.38 ± 0.60	−51.76 ± 0.74	−92.15 ± 0.71	44.92 ± 1.00	−47.23 ± 0.98

∆E_vdW_ = van der Waals energy; ∆E_elec_ = electrostatic energy; ∆G_gas_ = gas phase free energy, ΔE_elec_ + ΔE_vdW_; ∆G_solv_ = solvation free energy; ∆G_bind_ = calculated total binding free energy.

## Data Availability

The data supporting the findings of this study are available within the paper and its Supplementary Information File. Additional raw datasets generated during the study are available from the corresponding author upon reasonable request.

## Supplementary Materials

The following supporting information can be downloaded at: https://www.mdpi.com/article/10.3390/ph18121857/s1, Reference [56] is cited in the supplementary materials. Figure S1: The chemical structures of all 23 tested FDA-approved drugs tested in this study; Figure S2: phosphonamidite prodrugs; Figure S3: Monocyclic nitrogenous drugs; Figure S4: Purine based drugs; Figure S5: (a) Fused heterocyclic drugs, (b) Biphenyl containing system; Figure S6: metabolic pathway for valganciclovir and valaciclovir; Figure S7: metabolic pathway for remdesivir and sofosbuvir. Table S1: List of antiviral drugs tested in this study with their mechanisms of action; Table S2: List of drugs with their metabolism.

## Abbreviations

The following abbreviations are used in this manuscript:

Mpox	Monkeypox
MPXV	monkeypox virus
PHEIC	public health emergency of international concern
WHO	World Health Organization
GISAID	Global Initiative on Sharing All Influenza Data
VIGIV	Vaccinia Immune Globulin Intravenous
CC50	Cytotoxicity Concentration
IC50	Half Maximal Inhibitory Concentration
SI	Selectivity Safety Index
MM/GBSA	Molecular Mechanics Energy Approach
MD	Molecular Dynamic
RMSD	Root-Mean-Square Deviation
RMSF	Root-Mean-Square Fluctuation
RoG	Radius of Gyration
SASA	Solvent Accessible Surface Area
RdRp	RNA-dependent RNA polymerase
DMEM	Dulbecco’s Modified Eagle Medium
DMSO	Dimethyl Sulfoxide
CPE	Cytopathic Effect
TCID50	Tissue Culture Infection Dose
GAFF	General Amber Force Field
NPT	Isobaric-Isothermal Ensemble
GSA	Non-Polar Solvation Free Energy
GGB	Polar Solvation
