# Supplementary material for: Repurposing of FDA-Approved Antiviral Drugs Against Monkeypox Virus: Comparative In Vitro Screening and Structure Based In Silico Studies
Source: Pharmaceuticals (Basel). 2025 Dec 5;18(12):1857. doi: 10.3390/ph18121857 (PMC12736150; doi:10.3390/ph18121857)
Supplement: Supplementary file 1 [file pharmaceuticals-18-01857-s001.zip › pharmaceuticals-3972228-supplementary.pdf]

Repurposing of FDA-approved antiviral drugs against Monkeypox virus: Comparative in vitro screening and structure based in silico studies: Supplementary files.

Table S1. List of antiviral drugs tested in this study with their mechanisms of action.

Drug	Mechanism of action
**Abacavir**	Nucleoside analog reverse-transcriptase inhibitor
**Acyclovir**	DNA polymerase inhibitor
**Amantadine**	Antagonism of the influenza virus A M2 proton channel
**Chloroquine**	Increase late endosomal and lysosomal pH
**Daclatasvir**	HCV protein NS5A inhibitor
**Dolutegravir**	HIV integrase inhibitor
**Entecavir**	Deoxyguanosine analog reverse-transcriptase inhibitor
**Favipiravir**	Nucleoside analog RNA-dependent RNA polymerase inhibitor
**Hydroxychloroquine**	Interferes with endocytosis
**Lamivudine**	Cytidine analogue, reverse transcriptase inhibitor
**Molnupiravir**	Introducing copying errors during viral RNA replication
**Nevirapine**	Non-nucleoside reverse transcriptase inhibitor (NNRTI)
**Oseltamivir**	A neuraminidase inhibitor
**Penciclovir**	Guanosine analogue, viral DNA polymerase inhibitor
**Remdesivir**	Adenosine nucleoside triphosphate analog, RNA-dependent RNA polymerase inhibitor
**Ribavirin**	Guanosine analog that can be incorporated into viral RNA during replication
**Sofosbuvir**	NS5B inhibitor
**Tenofovir**	Nucleotide reverse transcriptase inhibitor
**Valaciclovir**	DNA polymerase inhibitor
**Valganciclovir**	A prodrug for ganciclovir, which is a synthetic analog of 2′-deoxy-guanosine.
**Velpatasvir**	NS5A inhibitor
**Zanamivir**	A neuraminidase inhibitor
**Zidovudine**	Thymidine analogue, reverse transcriptase inhibitor

Repurposing of FDA-approved antiviral drugs against Monkeypox virus: Comparative in vitro screening and structure based in silico studies: Supplementary files.

Table S2. List of drugs with their metabolism [1].

	**Metabolism**
**Penciclovir**	phosphorylated to penciclovir triphosphate (the active metabolite) in infected host cells
**Tenofovir**	phosphorylated to monophosphate (nucleotide), then _tenofovir diphosphate.
**Nevirapine**	Oxidative metabolism in the liver by cytochrome p450 isoforms CYP34A4 and CYP2B6 .
**Zidovudine**	Glucuronidation in the liver.<br>Also metabolized in renal microsomes (GAMT). Intracellular (host cell) conversion of zidovudine to the triphosphate derivative is necessary for the antiviral activity
**Lamivudine**	metabolite to the trans-sulfoxide by sulfotransferases.
**Daclatasvir**	substrate of CYP3A enzymes where its metabolism is predominantly mediated by CYP3A4 isoform.
**Abacavir**	Metabolized in liver. Abacavir is not significantly metabolized by cytochrome P450 enzymes.
**Entecavir**	Entecavir is efficiently phosphorylated to the active triphosphate form.
**Velpatasvir**	Some metabolisms by CYP2B6, CYP2C8, and CYP3A4.
**Sofosbuvir**	Sofosbuvir was cleaved by CatA and CES1 and subsequent activation steps included amino acid removal by histidine triad nucleotide-binding protein 1 (HINT1) and phosphorylation by uridine monophosphate-cytidine monophosphate (UMP-CMP) kinase and nucleoside diphosphate (NDP) kinase.
**Ribavirin**	ribavirin is phosphorylated intracellularly by adenosine kinase to ribavirin mono-, di-, and triphosphate metabolites.
**Favipiravir**	Favipiravir is extensively metabolized with metabolites excreted mainly in the urine. The antiviral undergoes hydroxylation primarily by aldehyde oxidase and to a lesser extent by xanthine oxidase to the inactive metabolite, T705M1.
**Acyclovir**	Acyclovir is becomes acyclovir monophosphate (due to the action of viral thymidine kinase) and diphosphate form (by guanylate kinase) then converted to acyclovir triphosphate (by nucleoside diphosphate kinase, pyruvate kinase, creatine kinase).
**Amantadine**	metabolites of amantadine have been identified in human urine.
**Zanamivir**	Not metabolized
**Oseltamivir**	converted to the active metabolite, oseltamivir carboxylate, by esterases located predominantly in the liver.
**Dolutegravir**	The first pathway by the glucuronidation, the second pathway by carbon oxidation by CYP3A4 and the third pathway is a sequential oxidative defluorination and glutathione conjugation. The main metabolite found in blood plasma is the ether glucuronide form (M2)
**Valaciclovir**	Valaciclovir is a DNA Polymerase Inhibitor.
**Valganciclovir**	Rapidly hydrolyzed in the intestinal wall and liver
**Hydroxychloroquine**	Hydroxychloroquine is N-dealkylated by CYP3A4 to the active metabolite desethylhydroxychloroquine, as well as the inactive metabolites desethylchloroquine and bidesethylchloroquine. Desethylhydroxychloroquine is the major metabolite.
**Remdesivir**	Remdesivir is a phosphoramidate prodrug that must be metabolized within host cells and **phosphorylated by cellular kinases to yield the active triphosphate form.**
**Chloroquine**	Chloroquine is N-dealkylated primarily by CYP2C8 and CYP3A4.
**Molnupiravir**	Molnupiravir exerts its antiviral action by introducing copying errors during viral RNA replication

Repurposing of FDA-approved antiviral drugs against Monkeypox virus: Comparative in vitro screening and structure based in silico studies: Supplementary files.

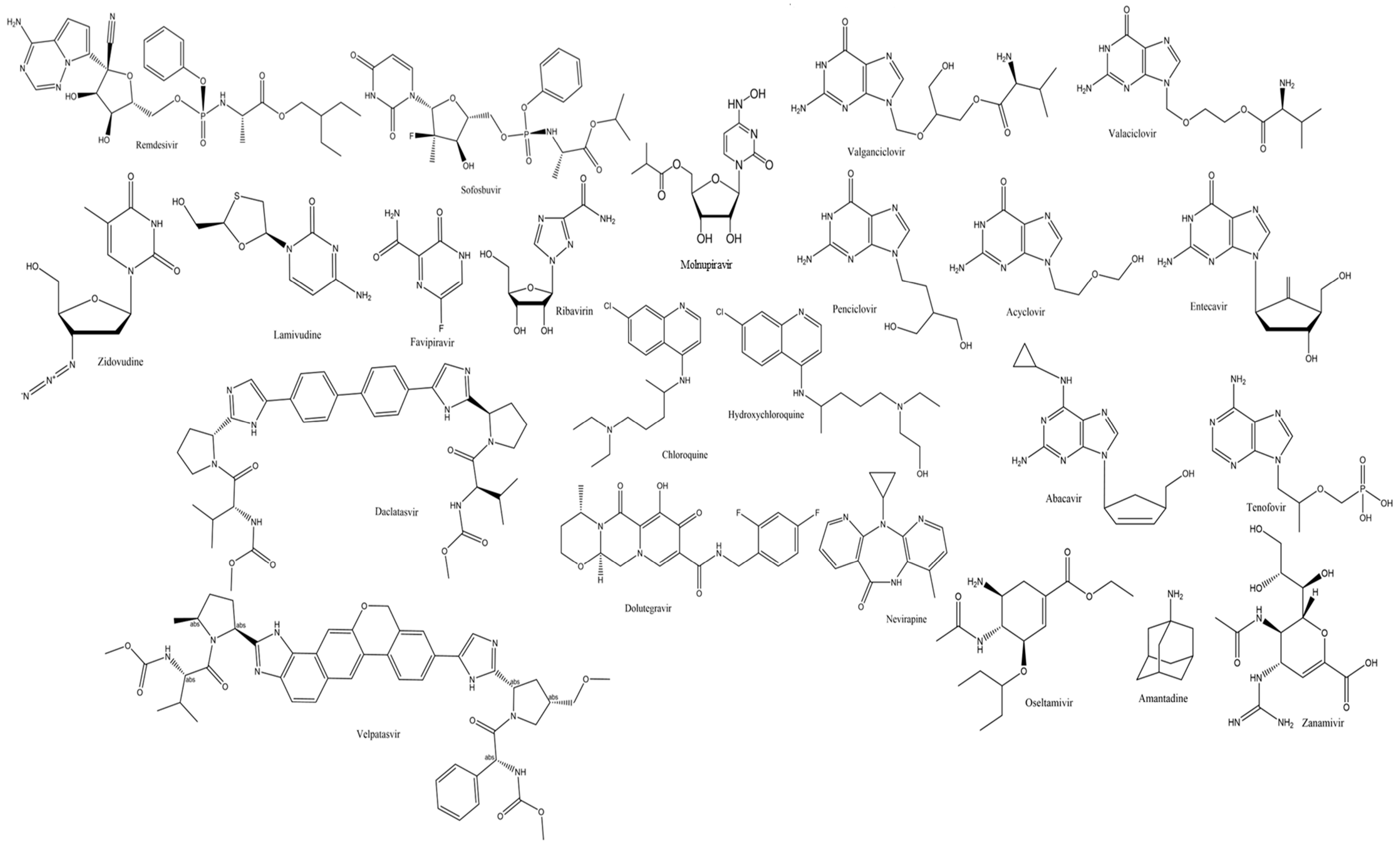

Figure S1. The chemical structures of all 23 tested FDA-approved drugs tested in this study.

Repurposing of FDA-approved antiviral drugs against Monkeypox virus: Comparative in vitro screening and structure based in silico studies: Supplementary files.

Drugs Classifications:

The tested antiviral drugs were categorized based on their chemical structu

res to identify their chemical descriptors, figures (S1-S4).

Classification of drugs based on their chemical structures

Most of these antiviral drugs belong to non-nucleoside family. However, we classified based on their core structure or main scaffold as

1- **phosphonamidite prodrugs**

These are nucleoside phosphate drugs that consisting of an amino acid ester linked via P-N bond to a nucleoside aryl phosphate. Such prodrugs have increased lipophilicity and thus are capable of altering cell and tissue distribution. The degree of hydrolysis of phosphonamidite functionality is apparent to be critical

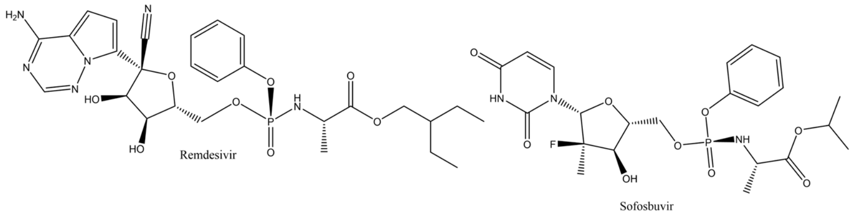

Figure S2. phosphonamidite prodrugs.

2- **Monocyclic nitrogenous drugs**

These are drugs contain substituted pyrimidine ring as zidovudine, and lamivudine or pyrazine ring as favipiravir or triazole ring as ribavirin.

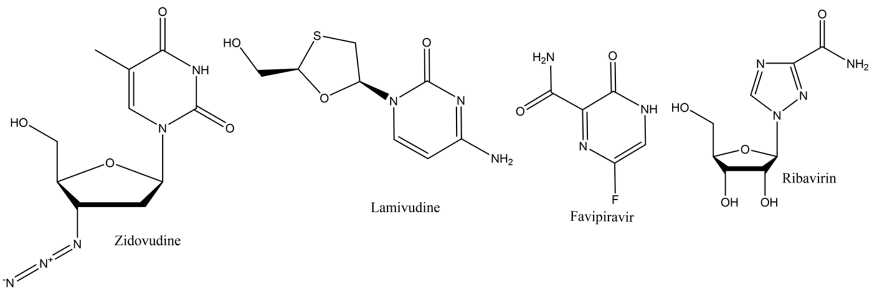

Figure S3. Monocyclic nitrogenous drugs.

Repurposing of FDA-approved antiviral drugs against Monkeypox virus: Comparative in vitro screening and structure based in silico studies: Supplementary files.

3- Purine based drugs

Guanine based skeleton

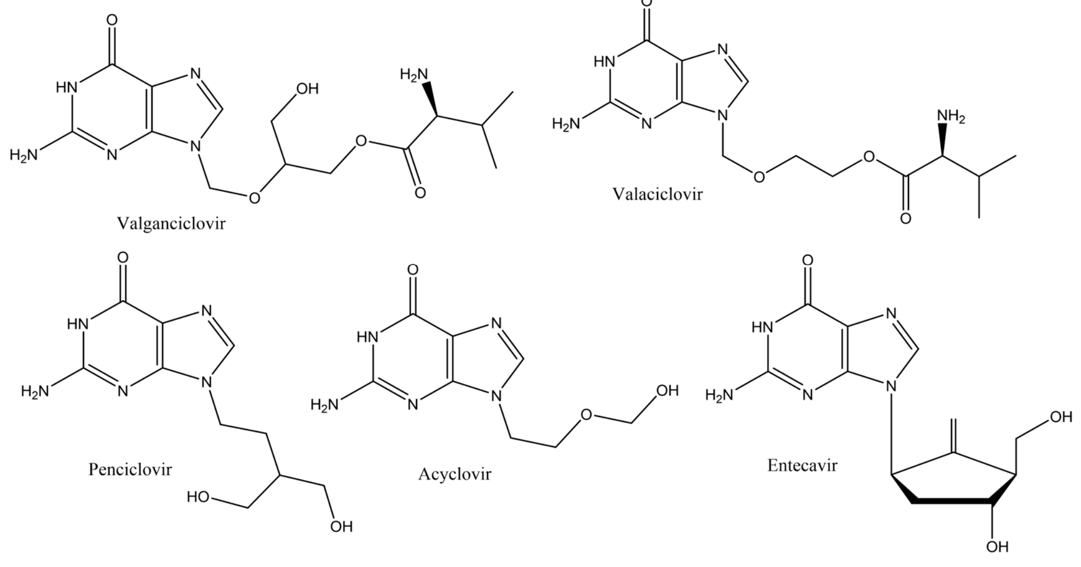

Carbocyclic drugs

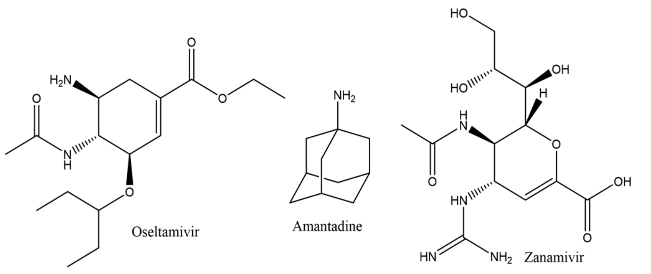

Adenine based skeleton

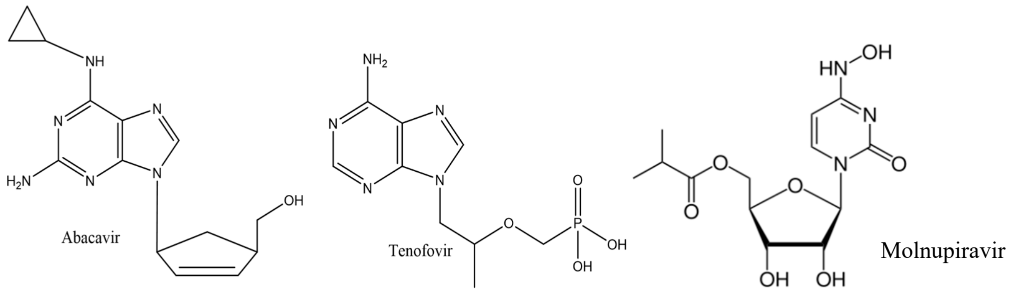

Figure S4. Purine based drugs.

Repurposing of FDA-approved antiviral drugs against Monkeypox virus: Comparative in vitro screening and structure based in silico studies: Supplementary files.

4- Fused heterocyclic drugs

a)

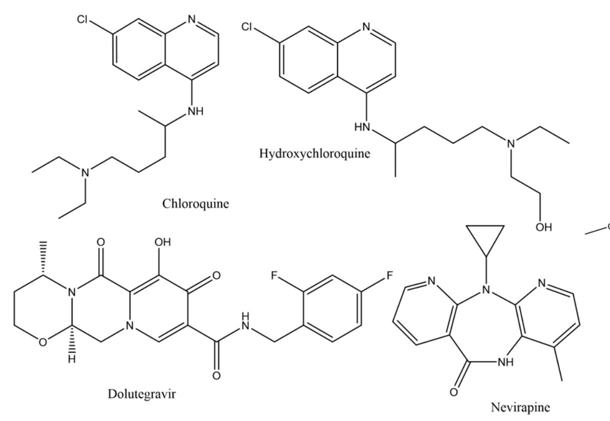

Fused heterocyclic drugs.

b)

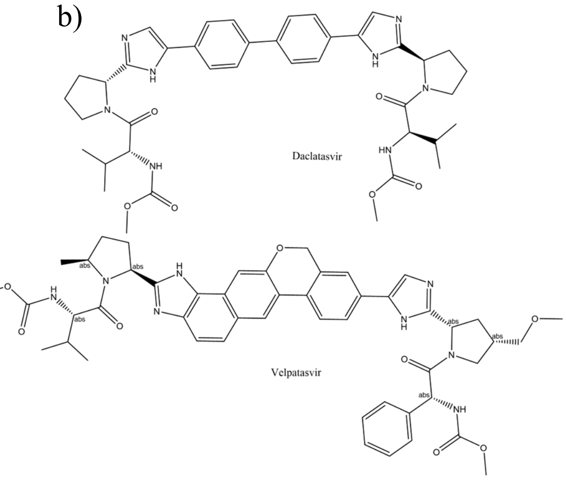

Biphenyl containing system

Figure S5. a) Fused heterocyclic drugs, b) Biphenyl containing system.

Repurposing of FDA-approved antiviral drugs against Monkeypox virus: Comparative in vitro screening and structure based in silico studies: Supplementary files.

Furthermore, to identify the role of pharmacodynamics of these drugs in this diversity of activity against selected mpox viruses, their reported metabolism of these drugs is listed table 1. It is likely most of these drugs undergo phosphorylation processes after hydrolysis as illustrated example in scheme 1 and 2 for two drugs from same category with different activities. *In vitro* activity, its is necessary for the antiviral activity of the most of drugs within the host cell. The ease and degradation of phosphorylation is critical for activity and shows the spot of the host ability to phosphorylate the drug.

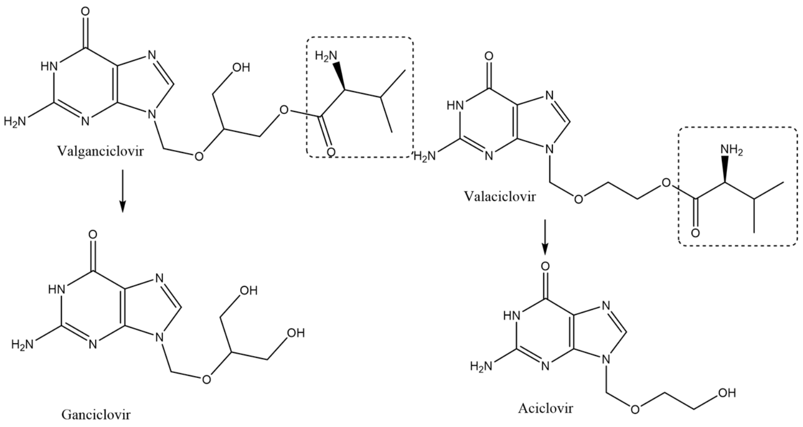

Figure S6. Metabolic pathway for valganciclovir and valaciclovir.

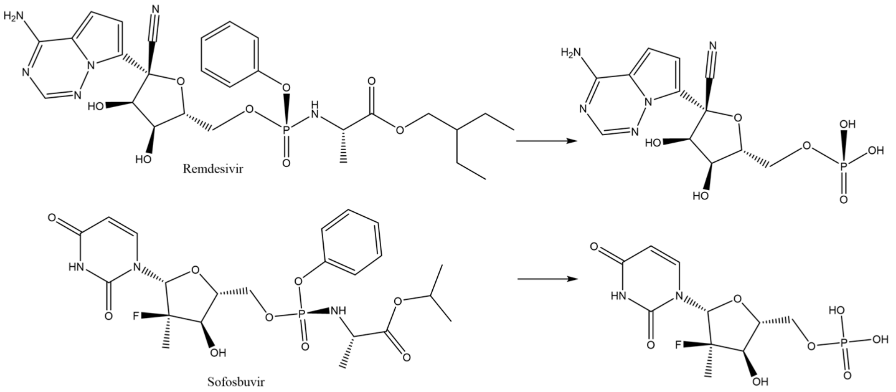

Figure S7. Metabolic pathway for remdesivir and sofosbuvir.

1. Law, V.; Knox, C.; Djoumbou, Y.; Jewison, T.; Guo, A.C.; Liu, Y.; Maciejewski, A.; Arndt, D.; Wilson, M.; Neveu, V.; et al. DrugBank 4.0: shedding new light on drug metabolism. *Nucleic Acids Res.* **2014**, *42*, D1091–D1097.
